# Looking Forward More Truly

**Published:** 1889-12

**Authors:** E. D. Babbitt

**Affiliations:** N. Y. College of Magnetics, 50 Union Square


### LOOKING FORWARD MORE TRULY.

BY E. D. BABBITT, M. D.

Mr. Preston thinks it strange that I "should become misled on this matter of population merely from reading Henry George." Two errors have crept into this statement: First, I have not been misled, and secondly, I have not formed my opinions from Henry George. In fact, I generally think for myself and differed from Mr. George even in that article. I totally differ from him in his one idea system of a single tax, but in many other respects he has masterly ideas concerning the rights of the people, and instead of being visionary, as Mr. Preston says, clinches his points with an immense weight of facts and principles.

Mr. Preston says he totally detests the teachings of Malthus. His article, however, was almost wholly on the Malthusian side, and gave fearful pictures of how fast we were tending toward destruction from the danger of over population. I showed that the world had been moving on through all the centuries and millenniums of the past, and yet was nowhere near being full The fact of the slow development of the Confucian family was a strong point, even if it should be proved that some other Chinamen develop more rapidly. Learned Chinamen say that the records of their country date back more than forty thousand years, but even after all this time, the country could sustain very many more people than it has, under a righteous system of distribution. China and India, like most other countries, have the most of their property in the hands of a few, while the millions are nearly on the verge of starvation. The danger, as I showed, is not from over population, but from over capitalization of the few, and almost starvation for the many. The minds of those who are alarmed for fear that human beings shall become as thick as locusts and eat each other up, I will attempt to relieve. Let us suppose that the estimate of Behm and Wagner, that the world's increase of population is 16,778,000 in nineteen months is correct, what then? Through science and improved machinery, men will be able to gain a great deal more out of mother earth than they now do, and will be able to sustain at least, in this way alone, a part of this increased population. After many thousand years, I think it quite probable that the earth will become full, at least as full as it ought to be to give every person refined and comfortable surroundings. Scores of centuries before that period, people will have been living together in that co-operative or mutual brotherhood which shall give each one culture, thoughtfulness and scientific self-control. Those who cannot see, under the light of to-day, that co-operation is to be the destiny of mankind, must have their perceptions and their humanitarian impulses well sealed up. Thousands of co-operative societies are already working harmoniously in Europe and are doing immense good.

At that era of the world when the race has evoluted into its marvelous wisdom, is it to be supposed that society will go on lawlessly increasing its offspring? I have not time or space here to state just how I think human offspring can be regulated so that the world will ever be kept full without *running over.* There is not the least cause for alarm, for evolution being true, the world's future must be a great deal happier and nobler than its past.

Mr. Preston says he will tell me something about Topolobampo Bay. I do not need to be told about it. I have studied its *pros* and *cons* for years through all kinds of channels. The Credit Foncier Colony that has gone there to carry out a beautiful system of social life which gives great individual liberty, combined with fraternal helpfulness, is one of the noblest bands of men and women on this planet. They had some severe struggles to get started, cut off from all communication with the rest of the world, as they have been, and they have been shamefully treated by a portion of the press, but the capital, as I learn, to complete their railroad and organize success, has been pledged. For any newspaper or correspondent to slur them or their work is to attack a divine cause and give new sorrows to those true souled people.

*N. Y. College of Magnetics*, 50 *Union Square.*

